# Early post-immobilization pain at rest, movement evoked pain, and their ratio as potential predictors of pain and disability at six- and 12-months after distal radius fracture

**DOI:** 10.1186/s40945-021-00101-6

**Published:** 2021-03-01

**Authors:** Maryam Farzad, Joy C. MacDermid, Saurabh Mehta, Ruby Grewal, Erfan Shafiee

**Affiliations:** 1 Department of Health and Rehabilitation Sciences, Roth McFarlane Hand and Upper Limb Centre, University of Western Ontario, School of Physical Therapy, St. Joseph’s Hospital, London, Ontario Canada; 2 Department of Occupational therapy, University of Social Welfare and Rehabilitation Sciences, Tehran, Iran; 3 Physical Therapy and Surgery, Western University, London, ON Canada; 4 Co-director Clinical Research Lab, Hand and Upper Limb Centre, St. Joseph’s Health Centre, London, Ontario Canada; 5 Rehabilitation Science McMaster University, Hamilton, ON Canada; 6 School of Physical Therapy, Marshall University, Huntington, WV USA; 7 Department of Orthopedic Surgery, Joan C Edwards School of Medicine, Marshall University, Huntington, WV USA; 8 Roth|McFarlane Hand and Upper Limb Center, Department of Surgery, Western University, London, ON Canada; 9 Department of Health and Rehabilitation Sciences, School of Physical Therapy, University of Western Ontario, London, Ontario Canada

**Keywords:** Chronic pain, Disability, Pain at rest, Movement, Evoked pain, Distal radius fracture

## Abstract

**Background:**

Removal of immobilization is a critical phase of distal radius fracture (DRF) rehabilitation, typically occurring by 2 months post injury. This study examined the extent to which pain at rest (PAR), movement evoked pain (MEP), or the ratio between those (MEPR) assessed at 2-months after DRF predicts the occurrence of chronic pain or disability at 6- and 12-months after the injury.

**Methods:**

This secondary analysis of a prospective cohort study was done at the Hand and Upper Limb Centre (HULC), London, Ontario, Canada. A total of 229 patients with DRF (159 (69.4%) women) were included. Scores for the pain and function subscales of the Patient-Rated Wrist Evaluation (PRWE) were extracted for 2, 6 and 12 months after DRF. Logistic as well as nonlinear quartile regression examined whether PAR and MEP predicted the severity of chronic pain and disability at 6- and 12-months after DRF. Receiver Operating Characteristics Curve were plotted, where area under the curve (AUC) examined the accuracy of the PAR and MEP scores in classifying those who experienced chronic pain and disability.

**Results:**

Scores of ≥3 (AUC of 0.77) for PAR or ≥ 6 (AUC of 0.78) for MEP at 2 months after DRF predicted moderate to severe wrist pain at 6-months, whereas scores of ≥7 (AUC of 0.79) for MEP at 2-months predicted ongoing wrist disability at 6-months after the injury. The MEPR of 2 ≤ or ≥ 8 at 2-months was associated with adverse pain at 6-months and functional outcomes at 6- and 12-months (R-square = 0.7 and 0.04 respectively), but prediction accuracy was very poor (AUC ≤ 0.50).

**Conclusion:**

Chronic wrist-related pain at 6-months can be predicted by either elevated PAR ≥ 3/10) or MEP (≥ 6/10) reported at 2-months after the injury, while disability experienced at 6-months after DRF is best predicted by MEP (≥7/10) reported at 2-months. The ratio of these two pain indicators increases assessment complexity and reduces classification accuracy.

## Introduction

Chronic pain is a common and disabling consequence of musculoskeletal (MSK) conditions [1]. The relationship between pain and movement is complex in these conditions, andmovement can mechanically aggravate pain or trigger inflammatory responses [2]. However, purposeful and gradual movements can enhance function without adversely affecting pain intensity [3]. Pain at rest (PAR) is typically the lowest level of pain and reflects the level of pain in an un-aggravated state. Movement evoked pain (MEP) reflects an assessment of pain intensity during movement, reflecting the extent to which pain is aggravated by movement. Both PAR and MEP are meaningful indices for patients and clinicians. PAR typically indicates the minimum level of pain that a patient is experiencing. Unacceptably high PAR indicates the need for better pain management [4]. It also indicates that pain is not arising from the actions of contractile structures and suggest that inflammatory/wound healing and/or central mechanisms may be contributing factors. MEP indicates the extent to which resting pain is aggravated by movement or the physiological processes involved in using contractile structures such as joint movement, soft tissue deformation, or muscle contraction. Movement is essential to performing prescribed exercises and daily activities of life. Pain with movement may contribute to fear of movement and reduced activity levels. For these reasons it might be theorized that MEP facilitates better understanding of functional disability in an individual with MSK injury and represents pain in context [2]. MEP is strongly influenced by peripheral mechanisms but can also be affected by central mechanisms [5].

Comparing PAR with MEP provides an assessment of irritability, which is the extent to which resting pain is worsened with movement. However, previous studies considering pain and disability predictors following hand injury have focused on overall pain scores without distinguishing PAR from pain with activity [6–8]. Further, a systematic review that identified the different traits of pain examined in clinical trials following surgeries such as total knee replacement (TKR) or thoracotomy indicated that most trials fail to differentiate specific traits such as PAR or MEP [4].

Previous trials have utilized global pain scales in determining overall pain experience using a combination of many different pain items, including assessment of PAR and MEP [4], where PAR and MEP are not analyzed separately. Emerging research in populations such as fibromyalgia [9] or those who undergo TKR for osteoarthritis of the knee [10] indicates that the MEP is an important prognostic indicator of chronic ongoing pain. However, the magnitude of MEP, its relationship with PAR, and its ability to characterize the risk of acute pain transitioning to chronic pain after hand injury have not been explored.

Distal radius fracture (DRF) is arguably the most common upper extremity fracture with a lifetime risk of 6.2 and 32.7%, respectively, in men and women who are 50-years of age [11]. While most patients recover well in the initial 6-months after DRF, 20–30% of individuals report ongoing pain and disability up to 2 years after DRF [12–14]. Since these fractures occur most commonly in middle-aged and older adults, persistent pain and disability can have an adverse impact on their ability to work, health behaviors, and overall participation in social or recreational activities [15]. Early identification of those at risk of developing persistent pain and disability following DRF has been advocated with promising early work elucidating the association between the severity of pain experience within two weeks after DRF and poor pain and functional outcomes over a longer term [8, 16]. Such early identification is purported to allow clinicians to implement different pain management strategies that would mitigate the risk of early intense pain experience and prevent patients from transitioning to chronic pain and disability. While this early research provides important insights, these studies have used composite pain rating scales to characterize early intense pain [8, 16].

It can also be argued that the intensity of pain examined within two weeks after DRF, while valuable, is not meaningful for physiotherapists managing patients with DRF. This is because most patients seek physiotherapy 6–8 weeks after the injury when the early immobilization required for acute fracture healing is over and the later mobilization and rehabilitation phase begins [17]. Therapists may not have access to pain intensity assessments conducted during the early fracture management phase. Therefore, it is essential to understand the associations between the pain experience assessed soon after mobilization of wrist is initiated in individuals with DRF and the longer-term occurrence of chronic pain and disability in these patients.

Performance-based tests of MEP have recently been described and used for predicting chronic pain outcomes in patients with neck pain [18, 19]. Such tests can also be developed and validated in patients with hand injuries. While these tests can be useful in research studies investigating clinical mechanisms and may be useful in applications on a limited scale, the time required to perform these tests makes them less desirable in high-volume outpatient practices catering to populations with MSK injuries. Further, performance-based tests that involve lifting heavy objects or repetitive movement might be contraindicated in the early post immobilization period after a fracture. However, many patients with fractures such as DRF transition from immobilization of wrist/hand to gradual mobilization, and start informal or formal physiotherapy, or hand therapy typically at 6–8 weeks following the injury. It is at this time point that clinicians are making decisions about exercise prescription for wrist mobilization, pain management strategies, or readiness of patient to resume injured wrist for functional and occupational use. Clinicians often ask about PAR and MEP during initial assessment to guide these aspects of clinical decision-making. If self-reported pain items on outcome measures administered during initial physiotherapy assessment can capture both PAR and MEP, they might provide more accurate prediction than unstructured interviews, single numeric pain ratings or composite pain rating scales that combine multiple pain indicators.

To advance understanding of prognosis following DRF, we evaluated whether certain pain behaviors, reported soon after wrist mobility is resumed, can identify individuals at risk of chronic pain and high disability after DRF. Our specific objective was to examine the ability of the scores for PAR as well as MEP obtained at 2-months after DRF, and the ratio of these two scores (MEPR) in predicting the occurrence of chronic pain and high disability at 6- and 12-months after DRF after adjusting for potential covariates. In addition, the study also aimed to identify scores on PAR, MEP, or MEPR obtained at 2-months after DRF that can accurately classify individuals at risk of chronic pain and high disability at 6- and 12-months after the injury should their predictive ability is confirmed.

## Methods

### Participants

The participants represented a subset of those who were recruited from the Hand and Upper Limb Centre, London, Ontario, Canada, between 1996 and 2009 as part of a cohort study. All the data for this prospective cohort were collected from the early mobilization period (2- months) to 12 months after the DRF injury. For the purposes of this study, we retrospectively included patients who provided data at their early post immobilization clinic visit (at 2-months) as well as at 6-months, and 12-months after the DRF. Prior research indicates that the majority of recovery occurs by 6-months, with smaller improvements up to 12-months after the injury [12]. This dataset did not contain information on fracture characteristics, acute-care pain management, and type orthopedic management. Most patients were referred for hand therapy consult at 6–8 weeks after injury irrespective of the nature of initial orthopedic management.

### Variables extracted

Data for demographic and injury variables and self-reported pain and disability ratings captured using Patient-Rated Wrist Evaluation (PRWE) at 2-months, 6-months, and 12- months after DRF were extracted from the dataset. The PRWE was developed for assessing outcomes of DRF, and the total score, subscales, and items have excellent reliability, content validity, and responsiveness in this population [20]. The pain subscale of the PRWE has an item-capturing PAR measured on a scale of 0–10, with 10 indicating the worst pain. Movement-evoked pain was defined as the mean score of 2 pain items that captured pain intensity during repeated wrist motion and while lifting heavy items. We also calculated the MEPR by dividing the score of MEP with the score on PAR. Of note, we had converted the scores on PAR and MEP from the scale of 0–10 to the scale of 1–11 (1 being no pain and 11 being the worst pain ever) to avoid divisions by 0 while calculating MEPR. This resulted in MEPR of 1 where pain with movement and pain at rest are equal and greater than 1 where movement results in higher pain level than PAR.

### Defining occurrence of adverse pain and high disability outcomes

Adverse pain outcomes were considered if patients continued to experience pain and disability at 6- and 12-months after DRF. Persistent moderate to severe pain was defined as a score of ≥12.5/50 on the pain scale of the PRWE [8]. We also defined severe chronic persistent pain at 6- and 12-months as a score of ≥35/50 on the pain scale of the PRWE. This threshold has been previously used to indicate chronic pain in the DRF population [8]. Similarly, persistent high wrist/hand disability at 6- and 12-months was defined as scores of ≥12.5/50 on the function scale of the PRWE [8].

### Statistical analysis

Demographic variables were described using mean and standard deviations (SD) for continuous variables and percentages for categorical variables. Normality of the continuous variables was examined using the Shapiro Wilk test [21].

Separate multivariate logistic regression models were created to examine the relationship between potential early post-mobilization pain characteristics and three different adverse 6 or 12-month outcomes in pain and disability (moderate to severe pain, severe pain, or disability). The models were adjusted for potential covariates such as age, sex, education level, injury to the dominant hand, energy level for fracture, being a smoker, presence of diabetes, and active claim for worker’s compensation for DRF. Odds ratios (ORs) were calculated with the 95% confidence interval (95% CI). Collinearity between these independent variables was examined where variance inflation factor of > 4 was considered indicative of significant collinearity in effect, rendering the regression analyses inaccurate [22]. *P* values of < 0.05 were considered indicative of significant associations. The adjusted R-square was used for assessing the predictive ability of the model. A curve quadratic regression model was used for examining the curve estimation for variables, which failed to fit in the linear model [23].

Receiver Operating Characteristic (ROC) curves were plotted to determine the accuracy of scores for PAR, MEP, and MEPR obtained at 2- months after DRF in classifying those who had adverse pain or disability outcomes at 6- and 12-months after DRF. The area under the curve (AUC) was estimated as an indicator of accuracy in classifying those who had an adverse outcome. We considered an AUC of 0.50 indicating no discriminative ability beyond chance, > 0.70 indicating acceptable accuracy, and > 0.80 indicating excellent accuracy [24]. Sensitivity and specificity values (SEN/SPE) were extracted from the ROC curves for each score interval for the PAR and MEP to determine the optimal cutoff scores at 2-months after DRF that best classified those with adverse pain and disability outcomes at 6- and 12-months after the injury.

## Results

A total of 229 patients who completed assessments at 2-months, 6-month, and 12-months following DRF were included in the study (Table 1). All the continuous variables satisfied the assumptions of normal distribution (*p* > 0.05), including PAR and MEP, with MEPR being the only exception. Table 1 shows the characteristics of the sample. Of these 229 participants, there were 159 (69.4%) women and 70 (30.6%) men. Women were significantly older compared to men (55 ± 14.7 years for women vs. 48.8 ± 15.5 years for men; *p* < 0.0001) in the cohort. There were no differences in the scores of PAR, MEP, or MEPR at 2-months, 6-months, or 12-months between men and women except for scores for PAR at 6-months where men reported higher pain compared to women (2.13 ± 2 for men vs. 1.76 ± 1.4; *p* < 0.003).

**Table 1:** Demographics and injury characteristics for the sample (*N* = 229)

		Women (***N*** = 159) Mean ± SD or %	Men (***N*** = 70) Mean ± SD or %	***P***-Value
Age		55 ± 14.7	48.8 ± 15.5	< 0.0001
Dominant Hand	R	147	58	14
	L	12	12	
Injured hand	R	63	25	0.55
	L	95	43	
	Both	1	2	
PAR - 2 M	2.72 ± 2.2	2.54 ± 2	0.63	PAR - 2 M
MEP - 2 M	6.32 ± 3	5.71 ± 2.7	0.07	MEP - 2 M
MEPR - 2 M	3.22 ± 2.3	3 ± 2.1	0.43	MEPR - 2 M
PAR - 6 M	1.76 ± 1.4	2.13 ± 2	0.003	PAR - 6 M
MEP - 6 M	3.87 ± 2.3	3.89 ± 2.7	0.68	MEP - 6 M
MEPR - 6 M	2.59 ± 1.5	2.28 ± 1.6	0.12	MEPR - 6 M
PAR - 12 M	1.51 ± 1.4	1.81 ± 1.7	0.07	PAR - 12 M
MEP - 12 M	3.11 ± 2.3	3.36 ± 2.8	0.36	MEP - 12 M
MEPR - 12 M	2.28 ± 1.4	2.04 ± 1.3	0.13	MEPR - 12 M

*SD* Standard Deviation, *R* Right, *L* Left, *PAR* Pain at Rest, *MEP* Movement Evoked Pain, *MEPR* Movement Evoked Pain Ratio

Table 2 summarizes the results of regression models with predictor variables shown for each adverse outcome at 6- and 12-months following DRF. Higher PAR scores at 2-months were significantly predictive of occurrence of moderate (OR, 1.92 [95% CI, 1.22 to 3.03]) and severe pain at 6-months (OR, 2.06 [95% CI, 1.09 to 3.91]) as well as moderate pain at 12-months after DRF (OR, 1.65 [95% CI, 1.14 to 2.39]). Apart from the association of lower education level with occurrence of moderate pain at 6-months after DRF (OR, 0.64 [95% CI, 0.42 to 0.97]), none of the other covariates were predictive of any other adverse outcomes at either 6- or 12-months following DRF. Higher MEP at 2-months were significantly predictive of occurrence of moderate pain (OR, 1.71 [95% CI, 1.18 to 2.46]) and disability (OR, 2.28 [95% CI, 1.18 to 4.42]) at 6-months after DRF. Apart from MEP, being a woman was also associated with occurrence of moderate pain (OR, 0.13 [95% CI, 0.02 to 0.83]) and disability (OR, 0.02 [95% CI, 0.001 to 0.43]) at 6-months after DRF. None of the other covariates were predictive of any other adverse outcomes at either 6- or 12-months following DRF.

**Table 2:** Linear regression Models for PAR and MEP at two months after DRF as independent variables (*N* = 229)

Outcome Variable		Adjusted R-Square	Odds Ratio	***P*** Values	95% CI
PAR	Moderate pain 6 m	0.50			
	PAR		1.92	0.005	1.22–3.03
	Education		0.64	0.035	0.42–0.97
	Moderate pain 1Y	0.44			
	PAR		1.65	0.008	1.14–2.39
	Severe pain 6 M	0.64			
	PAR		2.06	0.026	1.09–3.91
MEP	Moderate Pain - 6 Months	0.53			
	MEP		1.71	0.004	1.18–2.46
	Sex		0.13	0.031	0.02–0.83
	Disability - 6 Months	0.69			
	MEP		2.28	0.014	1.18–4.42
	Sex		0.02	0.02	0.001–0.43

*PAR* Pain at rest, *MEP* Movement evoked pain

Table 3 shows that a significant quadratic effect was seen between MEPR and the outcomes of pain and disability. Specifically, score of 2 ≤ or ≥ 8 for MEPR at two months after DRF were associated with pain at 6-months (OR, 0.88 [95% CI, 0.73 to 1.05]), disability at 6-months (OR, 0.74 [95% CI, 0.56 to 0.98]) and 12-months (OR, 1.05 [95% CI, 0.83 to 1.33]) after DRF.

**Table 3:** Non-linear quadratic regression models for Movement Evoked Pain Ratio at two months as independent variable (*N* = 229)

Outcome Variable	R-Square	Parameter Estimate b1	Parameter Estimate b2	Constant	*P*-value
**Pain - 6 Months**	0.46	−2.03	0.19	18.07	0.03
**Pain - 12 Months**	0.07	−0.24	−0.01	9.06	**0.65**
**Disability - 6 Months**	0.7	−0.3	0.21	13.98	0.02
**Disability - 12 Months**	0.04	−0.89	0.13	8.22	0.00

**Fig. 1:**
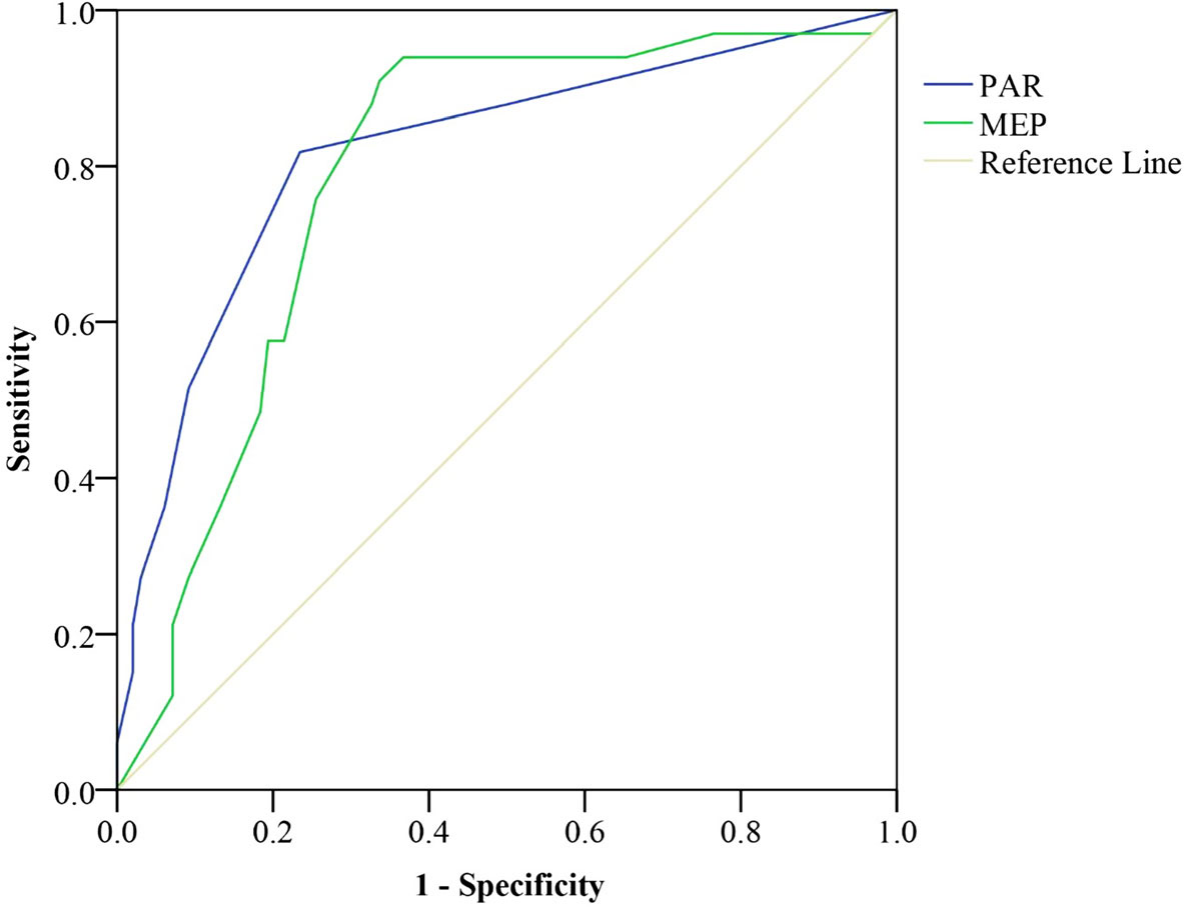
ROC curve of the PAR and MEP to predict 6-months disability; PAR: Pain at the Rest; MEP: Movement Evoked Pain

**Fig. 2:**
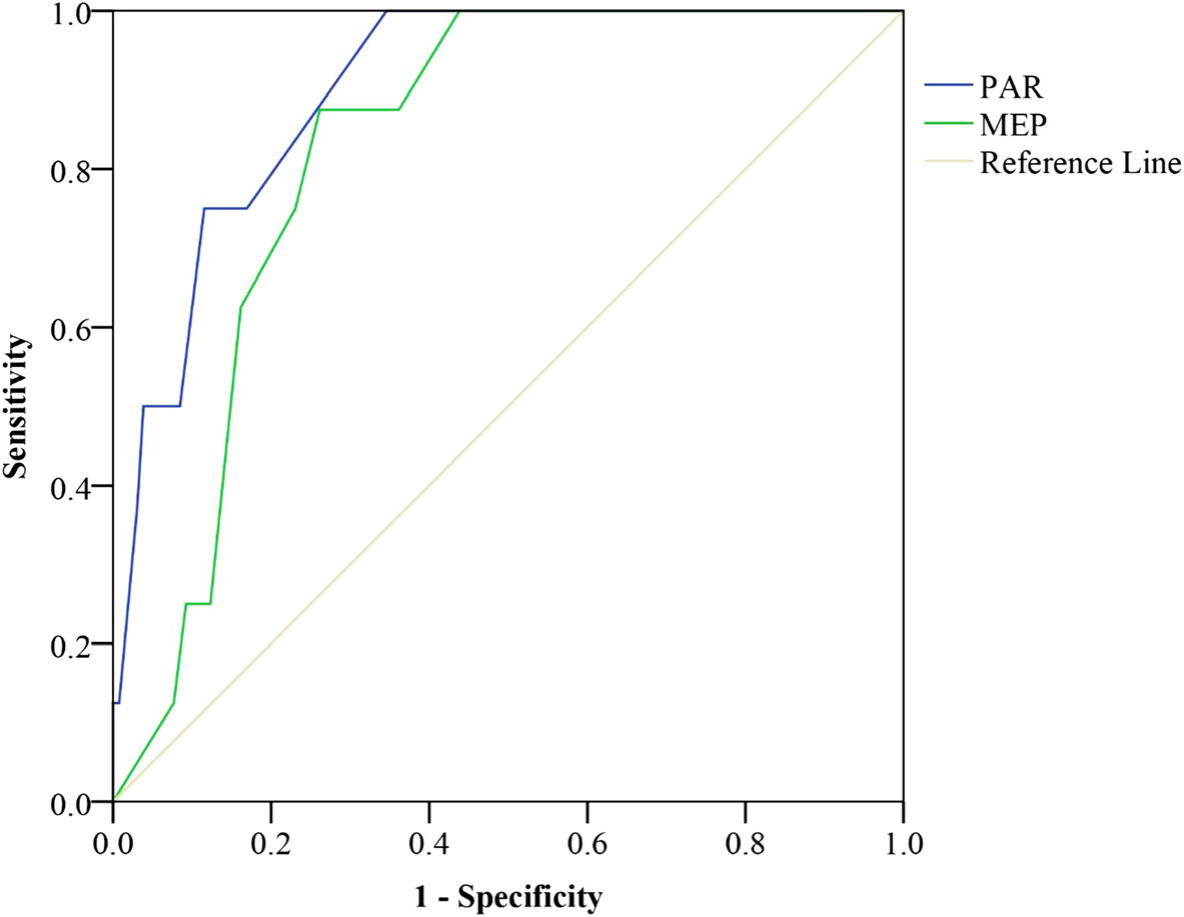
ROC curve of the PAR and MEP to predict 6-months moderate to severe pain; PAR: Pain at the Rest; MEP: Movement Evoked Pain

**Fig. 3:**
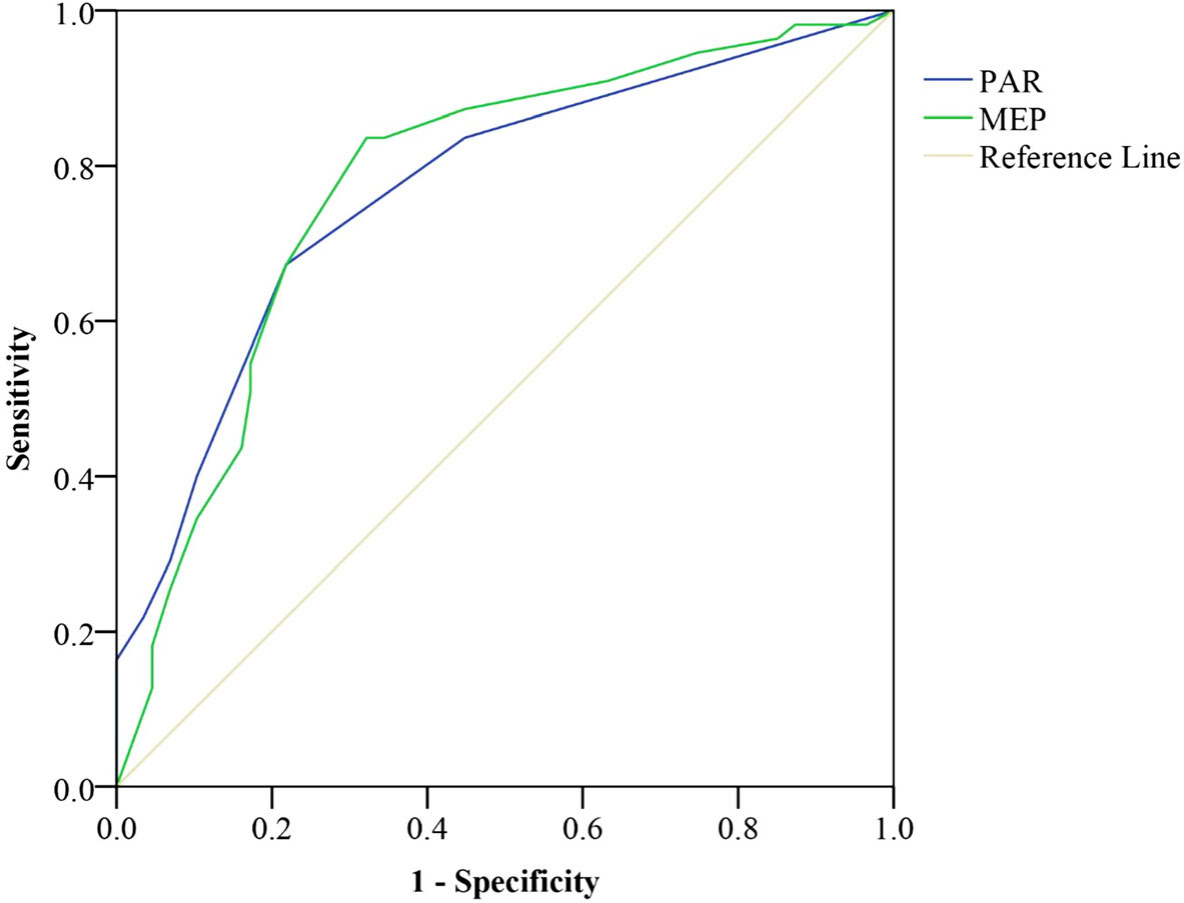
ROC curve of the PAR and MEP to predict 6-months severe pain; PAR: Pain at the Rest; MEP: Movement Evoked Pain

Figures 1, 2 and 3 show the ROC curves for 2-months scores of the PAR and MEP for adverse pain and disability outcomes at 6- and 12-months after DRF. The scores of ≥3 on PAR at 2-months after DRF were best able to classify those with moderate to severe pain (AUC 0.77, SEN/SPE 0.60/0.90), severe pain (AUC 0.90, SEN/SPE 0.75/0.88), and wrist/hand disability (AUC 0.82, SEN/SPE 0.51/0.91) at 6-months after DRF. Additionally, the scores of MEP of ≥6 at 2-months were best able to classify those with moderate to severe pain (AUC 0.78, SEN/SPE 0.67/0.79), and scores of ≥7 were best able to classify those with wrist/hand disability (AUC 0.79, SEN/SPE 0.58/0.81) at 6-months after DRF. The AUC values for MEPR scores at 2-months in classifying those with higher pain or disability at 6- and 12-months after DRF were low (AUC between 0.50–0.60). Therefore, AUC was not deemed an accurate predictor of pain and disability at 6- and 12-months after DRF.

In summary, a patient who reports ≥3 scores on PAR at 2-months after DRF is at risk of developing persistent, moderate, and severe pain at 6-months and moderate pain at 12-months after DRF. Scores of MEP of ≥7 at 2-months can predict disability at 6-months after injury.

## Discussion

Our results indicate that perceived pain intensity is an independent predictor of chronic pain after DRF, while pain with movement was is more predictive of disability. Specifically, individuals reporting pain intensity of ≥3 on PAR and ≥ 6 on MEP at 2-months after DRF are at high risk of experiencing moderate or severe pain at 6-, and 12 months following the injury. The higher PAR at 2-months is a stronger predictor of persistent pain, while MEP was a stronger predictor of disability at 6- and 12 months after DRF. Our research started with a hypothesis that the items of the PRWE that routinely measure PAR and MEP might be a useful indicator of condition irritability. While irritability is an important concept, the ratio of these two pain items was not a useful predictor of pain and disability over a longer term after DRF. We attribute this to unique aspects of using ratios.

Previous research has demonstrated that pain intensity obtained within 2-weeks of DRF is predictive of poor pain outcomes a year after DRF [8, 16, 25]. High pain levels in the initial few days of injury, when the wrist/hand is immobilized and joint movements are restricted, should indeed serve as a warning sign for clinicians. While this knowledge is useful, physiotherapists may not be involved in the case management at this early stage. We focused on the prediction based on scores from the early mobilization phase where patients would typically present to physiotherapy which occurs at approximately 6–8 weeks after the injury, when the fracture has sufficiently healed and immobilization devices are removed [26]. Knowledge concerning the associations between pain intensity reported during or within few days after initiating rehabilitation of DRF with poor pain and function outcomes after the injury has greater utility for physiotherapists. Such knowledge can enable physiotherapists to identify a risk profile for poor outcomes for an individual patient seeking rehabilitation of DRF. Since not every patient requires in-clinic supervised therapy and those with complications or at risk of adverse outcomes are more likely to require supervised rehabilitation after DRF [27], abilities to determine the risk profile for each patient can serve as an extremely useful clinical tool for physiotherapists in developing an individualized plan of care for each patient with DRF.

Our study showed that 2-months scores for PAR (score of ≥3/10), MEP (score of ≥6/10), and MEPR ≥8/10 are prognostic indicators of adverse outcomes after DRF. Previous research either used average pain intensity [16] or score on pain scales [8, 25] as prognostic indicators of chronic pain after DRF. Pain at rest and MEP are very useful pain indicators for understanding pain experience. High scores of PAR at 2- months after DRF, especially when the fracture has healed, can be one of the indicators of the presence of nociplastic pain or centrally mediated pain behavior [28], although a patient may have multiple pain mechanisms occurring simultaneously such as infection, hardware problems, and CRPS that might result in high pain. Further investigation would delineate whether patients have other indicators of centrally mediated pain that might require a therapy plan that target central pain [29], or other contributing factors. Conversely, MEP indicates irritability of pain with movement. Intense pain during movement might indicate to therapists that patients would be unlikely to complete their assigned therapeutic exercises which would inevitably a delay their recovery. Dynamic causes of pain including ligament, muscle or nerve injury, hardware issues, malalignment/union, or aggravation of underlying arthritis. Further examination would be required of these potential contributors. Movement evoked pain can also have psychological contributors and may contribute to development or reinforcement of fear avoidant behavior. This may explain why, movement-evoked was a better predictor of disability [30].

Using the cutoffs identified in this study for 2-months PAR (≥3/10) and/or MEP (≥7/10) will allow physiotherapists to consistently screen for risk of adverse outcomes after DRF, and will guide additional examinations which will support customized treatment plans. Having demonstrated that someone is presenting at elevated risk, physiotherapists will modify the initial assessment to look for potential physical and psychological factors that could be contributing to elevated pain. Depending on the findings of these examinations, therapist may implement modified treatment plans that target modifiable risk factors. Careful attention to the presence of edema and additional pain modalities, may be needed for those with higher pain experiences. Therapist may initiate cognitive reshaping type discussions with patients to align recovery expectations and educate patients in pain neuroscience [31]. Educating patients about the pain neuroscience (understanding of fear avoidance, pain catastrophization, patient expectations, cognitions, and beliefs) and explaining the relationships between pain and movement as they initiate physiotherapy for rehabilitation of DRF can reduce pain-related anxiety [32, 33]. Empowering patients to take an active role in their recovery and providing self-management skills for their pain can assist them with greater success in performing during therapeutic exercises and functional daily living tasks. Another approach is to progress wrist/hand exercises [34] in a graduated manner such that they optimize the recovery and minimize the setbacks that may arise from severe aggravation of pain, while providing greater self-efficacy [35] through accomplishment [36].

One of the other premises of this study was to examine whether MEPR, which is the ratio of MEP and PAR, could provide a useful indicator of the underlying irritability of the healing fracture. We conceptualized that the lower a MEPR, where pain with movement was similar to pain at rest would predict better recovery DRF. Physiotherapists commonly seek information about MEP and PAR during clinical examination. Pain irritability is a core construct in planning the intensity of rehabilitation programs and for progression.

Based on our results, the MEPR did not yield any meaningful information in identifying risk for persistent, moderate, or severe pain or poor wrist/hand function after DRF. Results suggested that 2- months MEPR scores, while showing some associations with pain and disability, did not discriminate between those who will or will not develop chronic pain at 6- and 12 months after DRF (AUC between 0.50–0.60). While irritability may be an important construct in assessing the impact of pain on overall function and calibrating exercise interventions, the MEPR ratio may not be the best measure to assess pain irritability. There are other reasons to support the finding that MEPR did not appear to have prognostic value in assessing the risk of poor outcome after DRF. Firstly, 0–10 scales for MEPR extracted from dividing two 0 to 10 scales (PAR and MEP) may be problematic from a measurement perspective. For example, a person with MEP score of 2/10 and PAR score of 1/10 is very different than someone who presents with PAR score of 5/10 and MEP score of 10/10, although in both cases the ratio would be same. Because most patient reported outcome measures are ordinal and not interval level data, mathematical operations can be problematic. Clinically, a doubling of pain when pain is at a very low level may not be clinically significant because it is still low; whereas, someone in moderate pain whose pain is doubled can be then in severe pain. Further, since the ratio is made up of two measures, each with its own source of measurement errors, computing the ratio may be subject to increased measurement errors. Finally, the scores for ratios such as the MEPR exist in a smaller range. Given these measurement considerations, we advise clinicians to evaluate irritability by considering PAR and MEP separately.

There are a few limitations of this study. Firstly, this was a retrospective cohort study where data was collected process prospectively but retrieved for the study analysis at a later date. This means that variables we may have analysed had they existed in the database such as fracture type, surgical management and use of pain medications were not present. These may have contributed additional explanatory power and/or modified the impact of the variables and models. However, since the information we did use was easily available to therapists upon, this information is most consistently able to be used by therapists in treatment planning. We did not have data on the nature of the rehabilitation services that patients received and so could not control for that. Our study did not include psychosomatic or behavioral factors, which are known to impact the recovery after DRF [37, 38] while assessing the associations of the PAR or MEP with chronic pain after DRF. Nonetheless, we believe that the impact of psychosomatic or behavioral factors on worse pain experience may have contributed to PAR and MEP scores. Therefore, using these scores for prognostication after DRF can be considered a comprehensive and simpler approach for physiotherapy practice. Secondly, we did not calculate the sample size because this was a retrospective analysis. However, post hoc analysis indicated we were sufficiently powered.

## Conclusions

The scores of ≥3 on PAR, and ≥ 6 on MEP at 2-months can predict long-term persistent pain after DRF. MEP (≥7/10) predicts a risk of disability at 6-months after injury. The scores of 2 ≤ MEPR≥8 can predict pain at 6- months and disability at 6- and 12 months after DRF. Clinicians may be able to use the cut point of patient-reported PAR, MEP, and MEPR items from the PRWE to screen individuals at risk of chronic pain and disability following DRF. The MEPR ratio may not be a useful indicator of pain irritability or future risk of pain and disability.

Data base with Ethic approval number of REB#5697 from Western Ontario university and Roth McFarlane Hand and Upper Limb Center, London, Ontario, Canada.

The prospective method and patient consent to participation is included in the ethic form.

## Data Availability

The datasets used and/or analyzed during the current study are available from the corresponding author on reasonable request.
